# Global perspectives on the burden of sexually transmitted diseases: A narrative review

**DOI:** 10.1097/MD.0000000000038199

**Published:** 2024-05-17

**Authors:** Chukwuka Elendu, Dependable C. Amaechi, Ijeoma D. Elendu, Tochi C. Elendu, Emmanuel C. Amaechi, Emmanuel U. Usoro, Nkechi L. Chima-Ogbuiyi, Divine B. Arrey Agbor, Chukwunnonso J. Onwuegbule, Eniola F. Afolayan, Benjamin B. Balogun

**Affiliations:** a Federal University Teaching Hospital, Owerri, Nigeria; b Igbinedion University, Okada, Nigeria; c Imo State University, Owerri, Nigeria; d Madonna University, Elele, Nigeria; e Babcock University, Ilishan-Remo, Nigeria; f Herzing University, Madison, WI; g Richmond University Medical Center, Staten Island, NY; h University of Ilorin, Ilorin, Nigeria.

**Keywords:** global burden, prevalence, prevention and control, public health, regional perspectives, sexually transmitted diseases, sociocultural factors

## Abstract

Sexually transmitted diseases (STDs) pose a significant global health challenge with far-reaching social, economic, and public health implications. These infections have haunted humanity from ancient times to today, transcending geographical boundaries and cultural contexts. This article explores the multifaceted landscape of STDs, delving into their epidemiology, pathophysiology, clinical manifestations, and global response strategies. The global prevalence of STDs is staggering, with millions of new cases reported annually. Prominent among these infections is HIV/AIDS, which remains a major global health crisis, affecting over 38 million people worldwide.

Additionally, bacterial STDs like chlamydia, gonorrhea, and syphilis continue to pose significant health risks, with millions of new cases reported yearly. Beyond the physical manifestations, STDs have profound social and economic implications. They can result in severe reproductive health issues, stigma, discrimination, and psychological distress, burdening healthcare systems and affecting individuals’ quality of life. The global response to STDs has been multifaceted, with international organizations and governments implementing various prevention and control strategies, including sexual education programs and scaling up access to testing and treatment. However, challenges persist, including disparities in healthcare access, sociocultural factors influencing transmission, and evolving pathogens with increasing resistance to treatment. Through case studies and real-world examples, we illuminate the human stories behind the statistics, highlighting the lived experiences of individuals grappling with STDs and the complex interplay of factors shaping their journeys. Ultimately, this review calls for continued research, innovative strategies, and sustained global commitment to mitigating the burden of STDs and promoting sexual health and well-being for all.

## 1. Introduction and background

Sexually transmitted diseases (STDs) represent a significant global health challenge with profound social, economic, and public health implications. These infections, caused by various bacterial, viral, and parasitic agents, are primarily transmitted through sexual contact but can also be transmitted vertically from mother to child during childbirth or through contaminated needles and blood products.^[1]^ Picture Maria, a 24-year-old college student from a rural community. Despite her cautious approach to sexual encounters, Maria finds herself grappling with the stigma and uncertainty of a positive chlamydia diagnosis. Her journey reflects the stark reality faced by millions worldwide, where the fear of judgment and lack of access to comprehensive sexual health services perpetuate the cycle of infection and silence. The global prevalence of STDs is alarmingly high, with millions of new cases reported yearly. According to the World Health Organization (WHO), it is estimated that over 1 million sexually transmitted infections (STIs) are acquired each day globally.^[2]^ Prominent among these infections is human immunodeficiency virus (HIV)/AIDS, which remains a major global health crisis, affecting over 38 million people worldwide.^[3]^ Consider the case of David, a 35-year-old man living in an urban center, who discovers he is HIV positive during a routine health checkup. His diagnosis shatters his world, confronting him with the reality of living with a chronic condition that requires lifelong management. David’s story highlights the devastating impact of HIV/AIDS on individuals, families, and communities, underscoring the urgent need for comprehensive prevention, testing, and treatment initiatives. Meet Ahmed, a migrant worker in a bustling urban center, illustrates the silent spread of hepatitis B among his community. His journey echoes the intersecting dynamics of migration, poverty, and healthcare access, illustrating the pervasive reach of STIs across diverse populations and settings.

Additionally, bacterial STDs like chlamydia, gonorrhea, and syphilis continue to pose significant health risks, with millions of new cases reported annually.^[4]^ The burden of STDs extends far beyond the physical health of individuals. It has profound social and economic implications. STDs can result in severe reproductive health issues, including infertility, ectopic pregnancies, and adverse pregnancy outcomes.^[5]^ Moreover, they can lead to stigma, discrimination, and psychological distress, often resulting in delayed diagnosis and treatment due to fear of social consequences.^[6]^ Economically, the cost of treating STDs and their complications is substantial. Direct medical expenses, including diagnostics, treatment, and follow-up care, burden healthcare systems considerably.^[7]^ The indirect costs related to lost productivity and reduced quality of life are substantial, particularly in resource-limited settings.^[8]^ The global response to STDs has been multifaceted. International organizations such as the WHO, UNAIDS, and UNICEF have launched comprehensive initiatives to combat these infections.^[9]^ National and regional governments have also implemented various prevention and control strategies, including sexual education programs, promotion of condom use, and scaling up access to testing and treatment.^[10]^ Despite these efforts, challenges persist, including disparities in access to healthcare resources, varying sociocultural factors influencing transmission, and evolving pathogens with increasing resistance to treatment. These challenges underscore the need for continued research, innovative strategies, and sustained global commitment to mitigating the burden of STDs.

## 2. Objectives of study

To Quantify the Global Burden of STDs using disability-adjusted life years (DALYs) and Standardized Metrics: This objective aligns closely with our enhanced methodology, which incorporates DALYs and other standardized metrics to quantify the health loss attributable to STDs. By applying rigorous epidemiological methods and data analysis techniques, we aim to provide a robust assessment of the overall burden of STDs, including morbidity, mortality, and disability, across different populations and geographic regions.

To Identify High-Risk Populations and Geographic Areas with Elevated STD Burden: Our review aims to go beyond merely quantifying the burden of STDs and seeks to identify vulnerable populations and geographic areas disproportionately affected by these diseases. By disaggregating data by age, sex, and geography, we can pinpoint areas with the highest disease burden and target interventions accordingly. This objective supports health planning efforts by guiding resource allocation and prioritizing interventions where they are most needed.

To Evaluate the Effectiveness of STD Prevention and Control Strategies: In addition to quantifying the burden of STDs, our review aims to assess the effectiveness of existing prevention and control strategies. We seek to identify best practices and gaps in current approaches by critically appraising the literature and analyzing intervention outcomes. This objective is crucial for informing health planning efforts and guiding the development of evidence-based interventions tailored to local contexts.

To Provide Evidence-Based Recommendations for Improving STD Prevention and Control Efforts: Our review aims to translate findings into actionable recommendations for improving STD prevention and control efforts globally. We can offer practical guidance for policymakers, healthcare practitioners, and public health officials by synthesizing and aligning the latest evidence with stakeholder priorities. These recommendations will support health planning efforts by promoting effective strategies, enhancing resource allocation, and advancing the global agenda for STD prevention and control.

## 3. Discussion

### 3.1. Methodology

To assess the global burden of STDs comprehensively, we employed a multifaceted approach that integrated various measures and indicators to quantify health loss and inform health planning efforts. Our methodology used DALYs as a primary metric, alongside other standardized indicators, to capture the full extent of morbidity and mortality attributable to STDs across different populations and geographic regions.

### 3.2. Measurement of health loss

DALYs served as a valuable tool for quantifying the overall burden of STDs by accounting for both years of life lost due to premature mortality and years lived with disability. By applying DALYs to STDs, we captured the impact of these diseases on individuals’ quality of life and mortality rates, providing a comprehensive understanding of their public health significance.

### 3.3. Data sources and age-sex breakdown

Data sources for our analysis included a combination of global health databases, epidemiological studies, and surveillance reports from reputable institutions such as the WHO, the Centers for Disease Control and Prevention (CDC), and academic research publications. These sources provided information on STD prevalence, incidence, and associated health outcomes.

To ensure a thorough analysis, we stratified the burden of STDs by age and sex, recognizing the differential impact of these diseases across different demographic groups. By disaggregating the data, we could identify vulnerable populations, such as adolescents, women of reproductive age, and men who have sex with men, who may bear a disproportionate burden of STD-related morbidity and mortality.

### 3.4. Geographic breakdown

Geographic variations in the burden of STDs were also explored, focusing on assessing disparities between regions and countries. We utilized global health maps and spatial analysis techniques to visualize the distribution of STD prevalence and incidence, highlighting areas with the highest disease burden and disparities in access to healthcare services.

### 3.5. Quality assurance and limitations

Quality assurance measures were implemented to ensure the reliability and validity of the data used in our analysis. Sensitivity analyses and validation checks were conducted to assess the robustness of our findings and address any potential biases or uncertainties.

While our methodology provided valuable insights into the global burden of STDs, it is essential to acknowledge certain limitations. These include variations in data availability and quality across different regions, underreporting of STD cases, and challenges in estimating the actual burden of asymptomatic infections. In addition to our comprehensive methodology for assessing the global burden of STDs, we have developed a practical algorithm aimed at enhancing clarity and applicability for health planning and intervention strategies. This algorithm, presented in Table 1, serves as a valuable tool for healthcare practitioners, policymakers, and public health officials to guide decision-making and resource allocation in preventing and controlling STDs.

**Table 1:** Practical algorithm for STD prevention and control.

Step	Action	Description
1	Surveillance and monitoring	Establish robust surveillance systems to monitor STD prevalence, incidence, and trends.
2	Risk assessment	Identify high-risk populations and geographic areas with elevated STD burden for targeted interventions.
3	Education and awareness	Implement comprehensive sex education programs to promote safe sexual practices and raise awareness about STD prevention.
4	Screening and testing	Offer routine STD screening for asymptomatic individuals, especially among high-risk groups. Ensure access to confidential and affordable testing services.
5	Treatment and management	Provide prompt diagnosis and appropriate treatment for individuals diagnosed with STDs. Ensure access to affordable medications and follow-up care.
6	Partner notification and contact tracing	Encourage individuals diagnosed with STDs to notify their sexual partners for testing and treatment. Conduct contact tracing to interrupt transmission chains.
7	Vaccination	Promote vaccination against vaccine-preventable STDs such as HPV and hepatitis B to reduce disease incidence and transmission.
8	Integration of services	Integrate STD prevention, testing, and treatment services with existing healthcare systems to improve access and utilization.
9	Community engagement	Engage communities and stakeholders in STD prevention efforts through outreach, education, and advocacy initiatives.
10	Evaluation and feedback	Regularly evaluate the effectiveness of STD prevention and control measures and adjust strategies based on feedback and surveillance data.

*Practical implementation*: This algorithm provides a step-by-step approach to addressing the burden of STDs and offers practical guidance for implementing evidence-based interventions. It emphasizes the importance of a comprehensive, multi-sectoral approach encompassing surveillance, education, screening, treatment, and community engagement.

*Visual enhancement*: Visual aids such as flowcharts or infographics can be incorporated to illustrate the sequential nature of the steps and facilitate comprehension to enhance the practicality of the algorithm. Additionally, explanatory notes can be included to provide further context and rationale for each action.

*Conclusion*: By integrating this practical algorithm into STD prevention and control efforts, stakeholders can streamline their approach, allocate resources more effectively, and ultimately reduce the burden of STDs on individuals and communities worldwide. This algorithm serves as a valuable resource for guiding evidence-based interventions and advancing the global agenda for STD prevention and control.

The practical algorithm presented in Table 1 offers a structured framework for addressing the challenges posed by STDs and underscores the importance of comprehensive prevention and control strategies. We encourage stakeholders to utilize this algorithm in their efforts to combat STDs and promote sexual health.

STDs = sexually transmitted diseases.

### 3.6. Epidemiology of STDs

STDs pose a significant global health burden, transcending geographical boundaries and socioeconomic strata. In a world increasingly interconnected through travel and technology, the prevalence and distribution of STDs have become intricate and multifaceted.^[11]^

Across the globe, an estimated 1 million individuals acquire an STD each day, underscoring the pervasive nature of these infections. While the burden is shared, regional variations in prevalence and incidence rates exist, reflecting disparities in healthcare access, education, cultural norms, and sexual behavior.^[12]^

In high-income countries, such as the United States and those in Western Europe, STD rates have witnessed a resurgence in recent years, driven by factors like decreased condom use, inadequate screening, and the emergence of drug-resistant strains. In contrast, low- and middle-income countries bear a disproportionate burden, grappling with challenges like limited healthcare infrastructure, stigma, and barriers to accessing prevention and treatment services.^[13]^

Disparities in age, gender, and sexual orientation mark the epidemiology of STDs. Young people aged 15 to 24 years are particularly vulnerable, accounting for a substantial proportion of new infections globally. Moreover, women, especially those in resource-limited settings, face heightened risks due to biological factors, gender-based violence, and limited agency in sexual decision-making.^[14]^

The advent of globalization has facilitated the spread of STDs across borders, with travel and migration serving as conduits for transmission. Urbanization further exacerbates this phenomenon, as densely populated areas create environments conducive to rapidly disseminating infections.^[15]^

Emerging trends, such as the intersection of STDs with other public health crises like HIV/AIDS and viral hepatitis, underscore the interconnectedness of health outcomes. Additionally, the advent of dating apps and online platforms has reshaped sexual networks, presenting both opportunities for prevention and challenges in tracing contacts and delivering care.^[16]^

### 3.7. Pathophysiology of STDs

At the heart of the pathophysiology of STDs lies a complex interplay between infectious agents and the human body’s defense mechanisms. STDs encompass diverse pathogens, including bacteria, viruses, parasites, and fungi, each with its unique transmission mode, pathogenesis, and clinical manifestations.^[17]^

Upon exposure to an infectious agent through sexual contact, the pathogen enters the body through mucosal surfaces, such as the genital tract, oral cavity, or rectum. The transmission efficiency varies depending on factors like pathogen virulence, host susceptibility, and predisposing factors such as genital trauma or inflammation.^[18]^

Once inside the body, the pathogen establishes infection by adhering to host cells, evading immune surveillance, and replicating within the local tissue. For bacterial STDs like gonorrhea and chlamydia, this process often involves colonization of the epithelial cells lining the genital tract, leading to inflammation, tissue damage, and the formation of characteristic clinical lesions.^[19]^

Viruses, such as herpes simplex virus (HSV), human papillomavirus (HPV), and HIV, employ diverse strategies to perpetuate infection and evade host defenses. HSV, for instance, establishes latency within the sensory ganglia following primary infection, periodically reactivating to cause recurrent episodes of genital ulceration and shedding.^[20]^ HPV, on the other hand, integrates into the host genome, predisposing to the development of genital warts and cervical, anal, and oropharyngeal cancers over time.

Parasitic STDs, including trichomoniasis and pubic lice infestation, thrive in the warm, moist environment of the genital region, where they reproduce and elicit local inflammation and irritation. Fungal infections, such as candidiasis, exploit disruptions in the vaginal microenvironment, such as alterations in pH or hormonal imbalances, to increase and cause symptomatic disease.^[21]^

The pathophysiology of STDs extends beyond local genital manifestations to encompass systemic sequelae and complications. Disseminated infections, ascending spread to the upper reproductive tract, and chronic inflammation can lead to severe consequences like pelvic inflammatory disease (PID), infertility, ectopic pregnancy, and an increased risk of HIV acquisition and transmission.

Furthermore, STDs can exert a profound impact on reproductive and maternal-child health outcomes, with implications for pregnancy outcomes, neonatal health, and vertical transmission of infections. For example, untreated syphilis during pregnancy can result in congenital syphilis, causing stillbirths, neonatal death, and a spectrum of congenital abnormalities.^[22]^

### 3.8. Types of STIs

STIs encompass a diverse array of pathogens, each with its unique characteristics, modes of transmission, and clinical presentations. From bacterial culprits to viral assailants and parasitic invaders, the world of STIs is as varied as it is, presenting global challenges to public health efforts.^[1]^

Bacterial STIs, such as gonorrhea and chlamydia, are notorious for their stealthy nature and propensity to cause silent infections, particularly in women. Despite their asymptomatic course in many cases, untreated bacterial STIs can lead to severe complications, including PID, infertility, and ectopic pregnancy. Neisseria gonorrhoeae, the bacterium responsible for gonorrhea, has exhibited alarming rates of antimicrobial resistance, posing a significant challenge to treatment efforts.^[2]^

Syphilis, caused by the bacterium Treponema pallidum, is a classic example of a bacterial STI with protean manifestations, progressing through distinct stages if left untreated. Primary syphilis presents as painless genital ulcers or chancres, while secondary syphilis manifests as a systemic illness with rash, fever, and mucocutaneous lesions.^[3]^ Tertiary syphilis, although rare in the era of antibiotics, can result in devastating cardiovascular and neurologic complications.

Viral STIs, including HSV, HPV, and HIV, exert a significant global health burden, with implications for morbidity, mortality, and quality of life. HSV infection presents with recurrent episodes of painful genital ulcers, accompanied by stigma and psychosocial distress. HPV, the most common viral STI worldwide, encompasses a multitude of genotypes, some of which are oncogenic and predispose to the development of cervical, anal, and oropharyngeal cancers.^[4]^

HIV/AIDS, perhaps the most formidable of all STIs, continues to take a heavy toll on individuals and communities worldwide. Despite advances in treatment and prevention, HIV/AIDS remains a significant public health challenge, with millions of new infections occurring annually. The virus not only undermines the immune system, leading to opportunistic infections and malignancies but also perpetuates social and economic inequalities, exacerbating the burden of disease among marginalized populations.^[5]^

Parasitic STIs, though less prevalent than their bacterial and viral counterparts, nonetheless pose significant health risks and challenges to control efforts. Trichomoniasis, caused by the protozoan parasite Trichomonas vaginalis, can cause vaginal discharge, itching, and discomfort, increasing the risk of HIV acquisition and transmission. Additionally, pubic lice infestation, or “crabs,” although more of a nuisance than a serious health threat, can cause itching and inflammation of the genital area.^[6]^

### 3.9. Clinical manifestations of STIs

STIs are notorious for their diverse and often elusive clinical presentations, ranging from asymptomatic carriage to debilitating symptoms and complications. The manifestations of STIs are influenced by factors such as the type of pathogen, site of infection, host immune response, and individual risk factors, making diagnosis and management challenging.^[1]^

Bacterial STIs, such as gonorrhea and chlamydia, can manifest as a spectrum of symptoms or remain entirely asymptomatic, particularly in women. When symptomatic, gonorrhea typically presents with urethritis, characterized by dysuria, urethral discharge, and, in some cases, epididymitis in men. Chlamydia infection, on the other hand, may cause urethritis, cervicitis, or PID, leading to lower abdominal pain, vaginal discharge, and dyspareunia.^[2]^

Syphilis, a systemic bacterial infection caused by Treponema pallidum, progresses through distinct stages if left untreated. Primary syphilis manifests as painless genital ulcers or chancres, often accompanied by regional lymphadenopathy. Secondary syphilis presents with a wide array of systemic symptoms, including rash, fever, sore throat, and mucocutaneous lesions.^[3]^ Tertiary syphilis, although rare in the era of antibiotics, can result in devastating cardiovascular and neurologic complications.

Viral STIs, such as HSV and HPV, are characterized by recurrent episodes of symptomatic disease interspersed with periods of asymptomatic shedding. HSV infection presents with painful genital ulcers or lesions, often accompanied by systemic symptoms such as fever, malaise, and lymphadenopathy. HPV infection, on the other hand, may cause genital warts, cervical dysplasia, or asymptomatic infection, depending on the viral genotype and host immune response.^[4]^

Hepatitis refers to liver inflammation, which various factors, including viruses, alcohol, drugs, toxins, and autoimmune diseases can cause. However, when discussing hepatitis in the context of STIs, the focus is primarily on viral hepatitis, specifically hepatitis B virus (HBV) and hepatitis C virus (HCV), which can be transmitted through sexual contact.^[5]^

HBV and HCV are bloodborne pathogens that can cause acute and chronic liver infections. While both viruses can be transmitted through exposure to infected blood, HBV can also be transmitted through sexual contact, sharing of contaminated needles, and from mother to child during childbirth. Sexual transmission of HBV is more likely to occur among individuals engaging in unprotected sex, particularly those with multiple sexual partners or individuals with a history of other STIs.^[6]^

Hepatitis B infection can manifest as acute or chronic hepatitis, with symptoms ranging from mild flu-like illness to severe liver damage, cirrhosis, and hepatocellular carcinoma (liver cancer). Acute hepatitis B infection may present with symptoms such as fatigue, jaundice (yellowing of the skin and eyes), dark urine, abdominal pain, and loss of appetite. Most adults with acute HBV infection recover completely, but a small percentage develop chronic hepatitis B, which can lead to long-term complications and liver damage if left untreated.^[7]^

Chronic hepatitis B infection is a significant cause of liver cirrhosis and liver cancer worldwide. It requires long-term management with antiviral medications to suppress viral replication and reduce the risk of disease progression. Additionally, vaccination against hepatitis B is highly effective in preventing new infections. It is recommended for individuals at increased risk of exposure, including sexually active adults, healthcare workers, and individuals with certain medical conditions.^[8]^

Hepatitis C virus (HCV) is primarily transmitted through exposure to infected blood, such as the sharing of contaminated needles, blood transfusions, or organ transplants. However, sexual transmission of HCV can also occur, particularly among individuals engaging in high-risk sexual behaviors such as unprotected anal intercourse, sex with multiple partners, and sex work. While the risk of sexual transmission of HCV is lower than that of HBV, it can still occur, especially in the presence of genital ulcers or mucosal trauma.^[9]^

Chronic hepatitis C infection is a leading cause of liver cirrhosis, liver failure, and hepatocellular carcinoma worldwide and requires early detection and treatment to prevent complications. Direct-acting antiviral medications have revolutionized the treatment of hepatitis C, offering high cure rates (>95%) with minimal side effects and shorter treatment durations. Screening for hepatitis C is recommended for individuals at increased risk of infection, including people who inject drugs, individuals with a history of blood transfusions or organ transplants, and those with high-risk sexual behaviors.^[10]^

HIV/AIDS, perhaps the most formidable of all STIs, can present with a spectrum of clinical manifestations, ranging from acute retroviral syndrome to advanced immunodeficiency and opportunistic infections. Acute HIV infection may mimic infectious mononucleosis, with symptoms such as fever, sore throat, rash, and lymphadenopathy. As the disease progresses, HIV/AIDS can cause opportunistic infections, malignancies, and neurologic complications, leading to significant morbidity and mortality if untreated.^[11]^

Parasitic STIs, although less common than bacterial and viral infections, can nonetheless cause significant morbidity and complications. Trichomoniasis, caused by the protozoan parasite Trichomonas vaginalis, presents with vaginal discharge, itching, and discomfort in women and urethritis in men. Pubic lice infestation, or “crabs,” manifests as itching and inflammation of the genital area, often accompanied by visible lice or nits.^[12]^

### 3.10. Uncommon STIs

Uncommon STIs may not be as well-known as their more prevalent counterparts. However, they still pose significant challenges to sexual health and require attention from healthcare providers and public health authorities. While less common, these infections can have severe consequences if left untreated and may be associated with unique clinical presentations, diagnostic challenges, and management considerations.

One example of an uncommon STI is lymphogranuloma venereum (LGV), a bacterial infection caused by specific serotypes of Chlamydia trachomatis. LGV primarily affects the lymphatic system, causing inflammation and swelling of the lymph nodes in the genital or anorectal region. Unlike other types of chlamydia infections, which typically present with urethritis or cervicitis, LGV may manifest as painful genital ulcers, tender inguinal lymphadenopathy (swollen glands), or proctitis (inflammation of the rectum). LGV can also lead to complications such as rectal strictures, fistulas, and genital elephantiasis if left untreated.^[1]^

Another uncommon STI is granuloma inguinale, also known as donovanosis, caused by the bacterium Klebsiella granulomatis. This infection is characterized by painless, beefy-red ulcers or nodules in the genital, perineal, or anorectal region, which can progress to extensive tissue destruction and disfigurement if untreated. Granuloma inguinale is rare in developed countries but remains endemic in certain regions, mainly tropical and subtropical areas with poor access to healthcare and sanitation.^[2]^

Additionally, sexually transmitted parasites such as Trichomonas vaginalis and Phthirus pubis (pubic lice) can cause uncommon STIs that may present with distinctive clinical features. Trichomoniasis, caused by the protozoan parasite T. vaginalis, can cause symptoms such as vaginal discharge, genital itching, dysuria (painful urination) in women and urethritis or prostatitis in men. While often asymptomatic, trichomoniasis can increase the risk of adverse pregnancy outcomes and HIV transmission if left untreated. Pubic lice infestation, or “crabs,” is characterized by intense itching and irritation in the genital area due to infestation with tiny parasitic insects that feed on human blood. While pubic lice infestation is typically considered a nuisance rather than a serious health threat, it can be sexually transmitted and may coexist with other STIs.^[3]^

Moreover, emerging STIs such as Mycoplasma genitalium and Zika virus have garnered attention in recent years due to their potential to cause reproductive complications and public health concerns. Mycoplasma genitalium is a bacterial infection associated with urethritis, cervicitis, and PID in women and urethritis and prostatitis in men. While less common than other STIs, Mycoplasma genitalium has been implicated in infertility, ectopic pregnancy, and adverse pregnancy outcomes. Zika virus, primarily transmitted through mosquito bites, can also be sexually transmitted, posing risks of congenital Zika syndrome (CZS) in pregnant women and neurological complications such as Guillain-Barré syndrome in adults.^[4]^

Bowenoid papulosis is a relatively uncommon STIcharacterized by small, reddish-brown papules or plaques on the genital skin or mucous membranes. This condition is caused by HPV infection, specifically high-risk strains such as HPV types 16 and 18, which are also associated with cervical and anal cancer.^[5]^

Bowenoid papulosis typically affects young, sexually active adults, particularly those who engage in unprotected sexual activity with multiple partners or have a history of genital warts. The lesions of Bowenoid papulosis are usually asymptomatic or may cause mild itching or irritation, leading some individuals to seek medical attention for cosmetic reasons or concerns about STIs.^[6]^

Clinically, the lesions of Bowenoid papulosis appear as raised, reddish-brown papules or plaques on the genital skin, shaft of the penis, vulva, or perianal area. The lesions may be solitary or multiple and can vary from a few millimeters to several centimeters in diameter. Unlike genital warts, which often have a characteristic cauliflower-like appearance, the lesions of Bowenoid papulosis are typically flat or slightly elevated and may have a smooth or rough surface.^[7]^

Diagnosis of Bowenoid papulosis is based on clinical examination and may be confirmed through biopsy and histopathological examination of the affected tissue. Histologically, Bowenoid papulosis is characterized by atypical keratinocytes (abnormal skin cells) with enlarged nuclei and increased mitotic activity, similar to the changes seen in precancerous lesions such as Bowen’s disease or squamous cell carcinoma in situ.

Management of Bowenoid papulosis typically involves conservative approaches such as topical treatments or observation, as most lesions spontaneously regress over time without causing complications. Topical treatments may include podophyllotoxin, imiquimod, or trichloroacetic acid (TCA), which can help reduce the size and appearance of lesions and promote resolution. Surgical interventions such as cryotherapy or laser therapy may be considered for persistent or symptomatic lesions, mainly if there is concern for malignant transformation or cosmetic reasons.

The prognosis for Bowenoid papulosis is generally favorable, with most lesions resolving spontaneously within months to years without causing long-term complications. However, individuals with Bowenoid papulosis should be counseled about the potential risk of HPV transmission to sexual partners and the importance of regular follow-up and screening for other HPV-related conditions, including cervical, anal, and oropharyngeal cancer. Additionally, vaccination against HPV can help prevent new infections and reduce the risk of HPV-related diseases, including Bowenoid papulosis.^[8]^

Finally, while uncommon STIs may not receive as much attention as more prevalent STIs, they still represent significant challenges to sexual health and require vigilance from healthcare providers and public health authorities. By raising awareness, improving diagnostic capabilities, and implementing targeted prevention and control strategies, stakeholders can work towards reducing the burden of uncommon STIs and promoting optimal sexual health for all individuals and communities.

### 3.11. Factors contributing to the global burden of STDs

The global burden of STDs is a multifaceted challenge shaped by an intricate interplay of biological, social, economic, and structural factors. At the heart of this burden lies a complex web of determinants, each contributing to the perpetuation and exacerbation of STDs on a global scale.

Biological factors play a pivotal role in driving the transmission and persistence of STDs. Pathogens such as bacteria, viruses, parasites, and fungi exploit vulnerabilities in the human body’s defenses, adapting and evolving to evade immune responses and antimicrobial therapies. Moreover, the intricate biology of STDs, including modes of transmission, incubation periods, and asymptomatic carriage, complicates efforts to control and prevent their spread.^[13]^

Social and behavioral factors significantly influence patterns of STD transmission and acquisition, reflecting the complex dynamics of human relationships, sexual practices, and cultural norms. Factors such as multiple sexual partners, inconsistent condom use, transactional sex, and substance abuse contribute to heightened risk behaviors and facilitate the spread of infections within and between communities.^[14]^ Moreover, stigma, discrimination, and lack of access to comprehensive sexual health education and services perpetuate misconceptions, hinder prevention efforts, and exacerbate the burden of STDs among marginalized populations.

Economic disparities and structural determinants of health further compound the global burden of STDs, exacerbating inequalities in access to prevention, screening, diagnosis, and treatment services. Limited healthcare infrastructure, inadequate funding for public health programs, and competing priorities divert resources away from STD control efforts, perpetuating cycles of transmission and disease burden.^[15]^ Moreover, poverty, unemployment, housing instability, and social marginalization create environments conducive to the spread of STDs, disproportionately impacting vulnerable populations and hindering efforts to achieve health equity.

Globalization and interconnectedness have facilitated the spread of STDs across borders, transcending geographical boundaries and exposing populations to novel pathogens and transmission dynamics. Travel, migration, and international trade contribute to the dissemination of infections, while urbanization and population mobility create environments conducive to the rapid transmission of STDs within densely populated areas. Furthermore, the emergence of digital technologies, including dating apps and online platforms, has reshaped sexual networks and behaviors, presenting both opportunities for prevention and challenges in reaching at-risk populations with targeted interventions.^[16]^

### 3.12. Global efforts in STD prevention and control

Efforts to prevent and control STDs on a global scale are characterized by a myriad of initiatives, partnerships, and strategies aimed at addressing the complex and multifaceted nature of these infections. From grassroots campaigns to multinational collaborations, the fight against STDs encompasses various stakeholders, interventions, and innovations.

At the forefront of global efforts in STD prevention and control are comprehensive sexual health education programs, which aim to empower individuals with knowledge, skills, and resources to make informed decisions about their sexual health.^[17]^ These programs promote healthy behaviors, encourage condom use, and foster open dialogue about consent, communication, and reproductive rights. By addressing underlying social norms and attitudes surrounding sexuality, sexual health education plays a crucial role in reducing risk behaviors and promoting positive sexual health outcomes.

Screening and testing initiatives form another cornerstone of STD prevention efforts, enabling early detection, diagnosis, and treatment of infections. Routine screening for STDs, particularly among high-risk populations such as sexually active adolescents, men who have sex with men, and individuals living with HIV, helps identify asymptomatic infections and prevent onward transmission. Furthermore, innovative approaches to screening, such as self-testing kits, mobile clinics, and community-based outreach programs, expand access to testing services and reach underserved populations with limited healthcare access.^[18]^

Treatment and management of STDs are integral components of comprehensive prevention and control efforts aimed at reducing morbidity, preventing complications, and interrupting transmission chains. Antimicrobial therapy, tailored to the specific pathogen and clinical presentation, is central to the management of bacterial STDs such as gonorrhea, chlamydia, and syphilis.^[19]^ Similarly, antiviral medications play a crucial role in the treatment of viral STDs such as HSV, HPV, and HIV, prolonging survival, improving quality of life, and reducing transmission risk.

Pre-exposure prophylaxis (PrEP) and post-exposure prophylaxis (PEP) offer additional strategies for preventing HIV transmission among high-risk individuals, including serodiscordant couples, injection drug users, and individuals with a history of condomless sex or STI diagnosis. PrEP, in particular, has emerged as a game-changer in HIV prevention, providing a highly effective biomedical intervention for individuals at risk of HIV acquisition. By combining antiretroviral medications with behavioral counseling and support services, PrEP programs offer a holistic approach to HIV prevention, addressing both biological and behavioral determinants of risk.^[20]^

Community engagement and advocacy efforts are essential for mobilizing resources, raising awareness, and fostering political commitment to STD prevention and control. Grassroots organizations, civil society groups, and advocacy networks play a crucial role in amplifying the voices of affected communities, advocating for policy change, and holding governments and policymakers accountable for addressing the needs of marginalized populations.^[21]^ By centering the experiences and priorities of those most affected by STDs, community-led initiatives help ensure that prevention and control efforts are equitable, inclusive, and responsive to the diverse needs of diverse communities.

Innovation and research drive progress in STD prevention and control, fostering the development of new technologies, interventions, and approaches to tackling the challenges posed by these infections. From novel diagnostic tests and treatment modalities to behavioral interventions and digital health solutions, innovation can revolutionize STD prevention and control, making services more accessible, efficient, and responsive to the evolving needs of populations worldwide.^[22]^

### 3.13. Regional perspectives on STDs

Across different regions of the world, the landscape of STDs varies significantly and is shaped by unique cultural, social, economic, and epidemiological factors. From high-income countries with well-established healthcare systems to low- and middle-income countries facing resource constraints, each region grapples with challenges and opportunities in preventing and controlling STDs.^[23]^

In high-income countries, such as those in North America, Western Europe, and parts of Asia-Pacific, STD control efforts are characterized by robust healthcare infrastructure, comprehensive sexual health education programs, and widespread access to screening, diagnosis, and treatment services. Despite these advantages, high-income countries face challenges such as increasing rates of antibiotic resistance, resurgence of syphilis and gonorrhea, and persistent disparities in access to care among marginalized populations, including ethnic minorities, LGBTQ + individuals, and immigrants.^[2]^

In contrast, low- and middle-income countries, particularly those in sub-Saharan Africa, South Asia, and Latin America, bear a disproportionate burden of STDs, driven by factors such as limited healthcare resources, poverty, gender inequality, and stigma. In these settings, challenges such as inadequate funding for STD control programs, shortages of trained healthcare personnel, and barriers to accessing preventive services hinder efforts to address the epidemic effectively. Additionally, cultural norms, taboos surrounding sexuality, and misconceptions about STDs contribute to delayed diagnosis, untreated infections, and ongoing transmission within communities.^[24]^

In regions affected by humanitarian crises, such as conflict-affected areas, refugee camps, and areas of natural disaster, the burden of STDs is exacerbated by disruptions to healthcare systems, displacement, and breakdowns in social support networks.^[25]^ Humanitarian organizations and non-governmental organizations play a crucial role in delivering essential sexual and reproductive health services, including STD prevention, testing, and treatment, to affected populations, often in challenging and resource-constrained environments.

In some regions, such as parts of Eastern Europe and Central Asia, injection drug use drives the spread of bloodborne infections, such as HIV and hepatitis C, highlighting the intersection of drug use, harm reduction, and infectious disease control. Harm reduction interventions, including needle exchange programs, opioid substitution therapy, and access to sterile injecting equipment, are essential components of comprehensive STD prevention efforts in these settings, helping to reduce transmission risk and improve health outcomes among people who inject drugs.^[26]^

Indigenous populations, particularly in regions such as Australia, Canada, and the Pacific Islands, face unique challenges in STD prevention and control, including cultural and linguistic barriers, historical trauma, and disparities in healthcare access. Culturally sensitive approaches that respect Indigenous knowledge, values, and traditions are essential for building trust, engaging communities, and addressing the underlying determinants of STDs effectively.^[27]^

### 3.14. Challenges and future directions in STD prevention and control

The landscape of STD prevention and control is marked by a dynamic interplay of challenges and opportunities, reflecting the complex nature of these infections and the evolving needs of populations worldwide. From persistent barriers to access and stigma to emerging threats such as antimicrobial resistance and technological advancements, stakeholders face a multitude of challenges in their efforts to curb the spread of STDs and improve sexual health outcomes.^[28]^

One of the foremost challenges in STD prevention and control is the persistent stigma and discrimination associated with these infections, which hinder individuals from seeking testing, treatment, and support. Addressing stigma requires multifaceted approaches that challenge misconceptions, promote empathy and understanding, and foster inclusive healthcare environments where individuals feel safe and supported to access sexual health services without fear of judgment or discrimination.^[29]^

Limited access to comprehensive sexual health education is another significant barrier to STD prevention, particularly among young people and marginalized populations. Effective sexual health education programs should go beyond mere anatomy and physiology to address topics such as consent, communication, healthy relationships, and contraception. By empowering individuals with accurate information and skills to make informed decisions about their sexual health, education programs can help reduce risk behaviors and promote positive sexual health outcomes.^[30]^

The rise of antimicrobial resistance poses a significant threat to the effectiveness of existing treatment regimens for bacterial STDs such as gonorrhea and syphilis. As pathogens evolve and develop antibiotic resistance, the arsenal of effective treatment options diminishes, exacerbating the challenge of managing and controlling these infections.^[31]^ To address this threat, stakeholders must prioritize antimicrobial stewardship, surveillance, and research into new treatment modalities, such as novel antibiotics and alternative therapies, to combat resistant strains effectively.

Emerging technologies, including telemedicine, mobile health applications, and point-of-care testing devices, offer promising avenues for expanding access to STD prevention and control services, particularly in remote and underserved areas. Telemedicine platforms enable individuals to access confidential consultations, testing, and treatment services remotely, reducing barriers to care and reaching populations who may face challenges accessing traditional healthcare settings. Similarly, mobile health applications can provide personalized sexual health information, reminders for testing and medication adherence, and support for behavior change, empowering individuals to take control of their sexual health in their own hands.^[32]^

Integrating STD services into primary care settings and other healthcare delivery platforms represents a promising strategy for expanding access to prevention, testing, and treatment services. By embedding sexual health services within existing healthcare infrastructure, such as family planning clinics, community health centers, and antenatal care programs, stakeholders can leverage existing resources, maximize efficiency, and reach individuals who may not otherwise seek out specialized STD services. Furthermore, integration facilitates comprehensive and holistic approaches to sexual health, addressing the full spectrum of reproductive and sexual health needs of individuals and communities.

### 3.15. Research and Innovation in STD prevention and control

In the ongoing battle against STDs, research and innovation serve as vital weapons, driving progress in prevention, diagnosis, treatment, and public health strategies. From groundbreaking discoveries in vaccine development to innovative technologies for screening and surveillance, the field of STD research is dynamic and multifaceted, characterized by continuous efforts to improve outcomes and address evolving challenges.

One area of significant progress in STD research is vaccine development, with efforts underway to develop safe and effective vaccines against viral pathogens such as HPV and HSV. HPV vaccines, in particular, have revolutionized cervical cancer prevention by targeting the strains responsible for the majority of cervical cancers and genital warts. Ongoing research seeks to expand vaccine coverage to additional HPV genotypes and explore the potential for therapeutic vaccines to treat existing infections and reduce transmission.^[33]^

Innovations in diagnostic technologies have also transformed STD prevention and control efforts, enabling rapid, accurate, and accessible testing for a wide range of pathogens. Point-of-care tests, molecular assays, and nucleic acid amplification techniques have revolutionized STD screening by providing results in real-time, facilitating timely treatment, and reducing the risk of onward transmission.^[34]^ Furthermore, advances in serological testing and biomarker discovery promise to improve early detection of infections and monitor treatment response.

Behavioral interventions and digital health technologies offer innovative approaches to promoting sexual health and preventing STD transmission among at-risk populations. Mobile health applications, social media platforms, and online interventions provide opportunities for delivering tailored sexual health information, promoting condom use, and facilitating partner notification and testing. Additionally, social network analysis and geospatial mapping tools help identify high-risk populations and target interventions where they are most needed.^[35]^

Advances in treatment modalities and antimicrobial therapies have expanded the options for managing bacterial and viral STDs, improving outcomes for affected individuals, and reducing transmission risk. Combination therapies, novel antibiotics, and alternative treatment regimens offer hope for addressing antimicrobial resistance and ensuring effective management of infections such as gonorrhea and syphilis. Similarly, antiviral medications and immune-based therapies hold promise for controlling viral STDs such as HIV and HSV, prolonging survival, and reducing the burden of disease.^[36]^

Epidemiological research and modeling play a crucial role in informing STD prevention and control strategies, providing insights into transmission dynamics, risk factors, and the impact of interventions. Mathematical modeling, phylogenetic analysis, and data visualization techniques help forecast disease trends, evaluate the effectiveness of prevention programs, and guide resource allocation decisions. Furthermore, collaborative research networks and global surveillance systems facilitate data sharing, standardization, and harmonization of methodologies, enabling cross-country comparisons and informing worldwide policy and programming.

### 3.16. Global partnerships and collaboration in STD prevention and control

In the fight against STDs, global partnerships and collaboration play a pivotal role in mobilizing resources, sharing knowledge, and coordinating efforts to address the complex challenges posed by these infections on a worldwide scale. From multinational organizations to grassroots initiatives, STD prevention and control is characterized by a web of interconnected stakeholders, each contributing their unique expertise, resources, and perspectives to the collective effort.

At the forefront of global STD prevention and control efforts are multinational organizations such as the WHO, UNAIDS, and the United Nations Population Fund (UNFPA). These organizations are vital platforms for coordinating international action, setting global health priorities, and mobilizing political commitment and financial resources to support STD control programs in low- and middle-income countries.^[37]^ Through initiatives such as the Global Fund to Fight AIDS, Tuberculosis, and Malaria and the President’s Emergency Plan for AIDS Relief (PEPFAR), these organizations provide funding, technical assistance, and capacity-building support to national governments and local partners to strengthen STD prevention and control efforts.

Regional partnerships and collaborations are crucial in tailoring STD prevention and control strategies to different regions’ unique epidemiological, cultural, and socioeconomic contexts. Regional organizations such as the European Centre for Disease Prevention and Control (ECDC), the Pan American Health Organization (PAHO), and the African Union (AU) facilitate information sharing, capacity building, and cross-border collaboration among member states to address common challenges and achieve shared health goals.^[38]^ Countries can leverage collective expertise and resources through regional networks to strengthen surveillance systems, harmonize standards and protocols, and implement evidence-based interventions responsive to local needs and priorities.

Non-governmental organizations (NGOs), civil society groups, and community-based organizations (CBOs) play a critical role in STD prevention and control efforts, particularly in reaching vulnerable and marginalized populations who may face barriers to accessing traditional healthcare services. These organizations provide frontline services such as HIV testing and counseling, condom distribution, and outreach to key populations such as sex workers, men who have sex with men, and people who inject drugs.^[39]^ Furthermore, NGOs and CBOs serve as advocates for policy change, human rights, and social justice, amplifying the voices of affected communities and holding governments and policymakers accountable for addressing the needs of those most at risk of STDs.

Academic institutions, research organizations, and public-private partnerships contribute valuable expertise and resources to STD prevention and control efforts, drive innovation, advance scientific knowledge, and translate research findings into practice. Collaborative research networks such as the HIV Prevention Trials Network (HPTN), the International AIDS Vaccine Initiative (IAVI), and the Global Antibiotic Research and Development Partnership (GARDP) facilitate interdisciplinary collaboration, data sharing, and capacity building to address priority research questions and develop new tools and strategies for STD prevention, diagnosis, and treatment.^[40]^

### 3.17. Policy and advocacy in STD prevention and control

In the realm of STD prevention and control, policy and advocacy efforts serve as powerful tools for driving change, shaping priorities, and promoting the rights and well-being of individuals and communities affected by these infections. From advocating for increased funding and resources to pushing for legislative reforms and challenging stigma and discrimination, policy and advocacy play a critical role in shaping the landscape of STD prevention and control at local, national, and global levels.

One of the primary goals of policy and advocacy in STD prevention and control is to mobilize political commitment and financial resources to support comprehensive and evidence-based interventions. Advocacy organizations, civil society groups, and affected communities play a crucial role in raising awareness about the importance of sexual health, highlighting the impact of STDs on individuals, families, and communities, and calling for increased investment in prevention, testing, treatment, and support services.^[41]^ By engaging policymakers, legislators, and government officials, advocates can influence budget allocations, policy decisions, and program priorities to ensure that STD prevention and control efforts are adequately resourced and prioritized within broader health agendas.

Policy advocacy also plays a crucial role in challenging discriminatory laws, policies, and practices that impede access to sexual health services and perpetuate stigma and discrimination against affected populations. Laws criminalizing HIV transmission, punitive regulations targeting sex workers and people who inject drugs, and discriminatory policies related to sexual orientation and gender identity hinder efforts to reach key populations with essential prevention and care services and fuel the spread of STDs. Advocates work tirelessly to repeal discriminatory laws, promote human rights and dignity, and create enabling environments that support access to comprehensive sexual health services for all individuals, regardless of their sexual orientation, gender identity, or social status.^[42]^

Moreover, policy advocacy efforts seek to promote evidence-based approaches to STD prevention and control that prioritize the needs and preferences of affected communities. Advocates push for policies and programs grounded in scientific evidence, respect human rights, and address the social determinants of health, including poverty, gender inequality, and structural violence. By championing approaches such as harm reduction, comprehensive sexuality education, and community-led interventions, advocates can help ensure that STD prevention and control efforts are effective, equitable, culturally sensitive, and responsive to the diverse needs and realities of different populations.^[43]^

International advocacy and diplomacy play a critical role in shaping global norms and standards for STD prevention and control, fostering collaboration among countries, and promoting best practices in policy and programming. Through forums such as the United Nations General Assembly Special Session (UNGASS) on HIV/AIDS, the International Conference on Population and Development (ICPD), and the World Health Assembly (WHA), advocates can influence global health agendas, advocate for the rights of affected populations, and hold governments accountable for their commitments to STD prevention and control. Furthermore, international partnerships and collaborations facilitate knowledge sharing, capacity building, and resource mobilization to support STD control efforts in low- and middle-income countries, where the burden of disease is often most significant.^[44]^

### 3.18. Intersectionality and STD prevention and control

In the realm of STD prevention and control, the principles of intersectionality and health equity are crucial for understanding and addressing the complex and intersecting factors that influence STD risk, vulnerability, and access to care. Intersectionality acknowledges that multiple intersecting factors, including race, ethnicity, gender, sexual orientation, socioeconomic status, immigration status, and disability, among others, shape individuals’ experiences of health and well-being.^[45]^ By recognizing diverse populations’ unique experiences and needs, intersectionality provides a framework for promoting health equity and addressing disparities in STD prevention and control efforts.

One key aspect of intersectionality in STD prevention and control is the recognition of how social determinants of health intersect with STD risk and vulnerability. Structural factors such as poverty, racism, sexism, homophobia, and discrimination contribute to disparities in access to sexual health information, resources, and services, exacerbating STD risk and limiting individuals’ ability to protect themselves from infection. For example, marginalized communities may face barriers such as lack of insurance coverage, transportation, or childcare, which hinder their ability to access STD testing, treatment, and prevention services. By addressing these structural inequities, stakeholders can create more equitable and inclusive healthcare systems that ensure all individuals have access to the resources they need to maintain sexual health and well-being.^[46]^

Moreover, intersectionality highlights the importance of recognizing and addressing the unique needs and experiences of key populations who face disproportionate burdens of STDs. Groups such as racial and ethnic minorities, LGBTQ + individuals, sex workers, people who inject drugs, incarcerated individuals, and adolescents may experience heightened vulnerability to STDs due to a combination of social, economic, and structural factors, including stigma, discrimination, violence, and marginalization. By centering the experiences and priorities of these populations, stakeholders can develop culturally competent and responsive interventions that meet their specific needs and promote health equity.^[47]^

Intersectionality also underscores the importance of taking a holistic and comprehensive approach to STD prevention and control that addresses the intersecting social, economic, and health-related needs of individuals and communities. This approach recognizes that STDs do not occur in isolation but are intertwined with broader issues such as poverty, housing instability, mental health, substance use, and access to education and employment.^[48]^ By integrating STD services with other essential health and social services, stakeholders can address the root causes of health inequities and promote holistic well-being among marginalized populations.

Furthermore, intersectionality emphasizes the importance of engaging affected communities in designing, implementing, and evaluating STD prevention and control efforts. Community-led approaches that prioritize participatory decision-making, cultural humility, and mutual respect empower individuals and communities to take ownership of their sexual health and advocate for their needs. By building partnerships with community-based organizations, grassroots activists, and affected individuals, stakeholders can ensure that STD prevention and control efforts are responsive to local contexts, culturally appropriate, and effective in addressing the needs of diverse populations.

### 3.19. Community engagement and empowerment in STD prevention and control

Community engagement and empowerment are fundamental principles in the realm of STD prevention and control, emphasizing the importance of involving affected communities as active partners in identifying priorities, shaping interventions, and driving change. By recognizing the expertise, knowledge, and resilience of communities and fostering collaboration and shared decision-making, community engagement, and empowerment efforts aim to build trust, strengthen resilience, and promote sustainable solutions to the complex challenges of STDs.

At the heart of community engagement and empowerment is recognizing that communities affected by STDs are not passive recipients of services but active agents of change with unique insights and experiences to contribute. By engaging affected communities in the design, implementation, and evaluation of STD prevention and control efforts, stakeholders can ensure that interventions are responsive to local needs and culturally appropriate and effectively address the root causes of STD transmission. Community-based organizations, grassroots activists, and peer educators are crucial in mobilizing community members, raising awareness, and advocating for policies and programs that promote sexual health and well-being.^[49]^

Community engagement involves various activities to build trust, foster dialogue, and promote collaboration among diverse stakeholders. Community forums, town hall meetings, and focus groups allow community members to voice their concerns, share their experiences, and contribute ideas for addressing STD-related challenges. Similarly, participatory research approaches, such as community-based participatory research (CBPR), involve community members as active participants in the research process, empowering them to shape research questions, methods, and outcomes and ensuring that research findings are relevant and actionable.^[50]^

Empowerment is central to community engagement efforts, emphasizing the importance of building the capacity of communities to advocate for their needs, mobilize resources, and drive change. Empowerment approaches focus on strengthening individual and collective skills, knowledge, and self-efficacy through education, training, and leadership development initiatives.^[51]^ By providing opportunities for community members to build confidence, assertiveness, and advocacy skills, stakeholders can empower communities to challenge stigma, discrimination, and social inequities that fuel the spread of STDs and advocate for policies and programs that promote sexual health and rights.

Moreover, community engagement and empowerment efforts prioritize the inclusion of marginalized and vulnerable populations who may face intersecting barriers to accessing sexual health services and information. By centering the voices and experiences of groups such as racial and ethnic minorities, LGBTQ + individuals, sex workers, people who inject drugs, and adolescents, stakeholders can ensure that interventions are equitable, inclusive, and responsive to the diverse needs of diverse communities.^[51]^ Culturally competent approaches that respect diversity, foster trust, and build partnerships with community-based organizations are essential for reaching marginalized populations and addressing disparities in STD outcomes.

### 3.20. Surveillance and monitoring

Surveillance and monitoring are vital components of STD prevention and control efforts, providing essential data and insights into the epidemiology, trends, and impact of STDs on populations worldwide. By systematically collecting, analyzing, and disseminating information on STD prevalence, incidence, risk factors, and outcomes, surveillance systems enable stakeholders to track disease trends, identify emerging threats, target interventions, and evaluate the effectiveness of prevention and control strategies.^[52]^

One key aspect of STD surveillance is the monitoring of disease burden and trends over time. Surveillance systems collect data on the number of reported cases, prevalence rates, and demographic characteristics of affected individuals, providing insights into the distribution and dynamics of STDs within and between populations.^[53]^ Trends in STD incidence and prevalence can reveal patterns of transmission, identify high-risk populations, and inform targeted prevention and control efforts in communities where STD rates are highest. Furthermore, longitudinal surveillance data enable stakeholders to monitor the impact of interventions, track progress towards national and global targets, and identify areas where additional resources and support may be needed.

Surveillance systems also play a crucial role in detecting outbreaks and clusters of STDs, facilitating timely responses to emerging threats, and preventing further transmission within communities. By monitoring trends in STD diagnoses, test positivity rates, and geographic distribution, surveillance systems can identify clusters of cases, detect changes in transmission patterns, and alert public health authorities to potential outbreaks.^[32]^ Early detection of outbreaks enables stakeholders to implement targeted interventions such as enhanced screening, contact tracing, and community outreach to control transmission and prevent further spread of STDs.

In addition to monitoring disease burden and trends, STD surveillance systems track key risk factors and determinants of STD transmission, providing insights into the social, behavioral, and environmental factors that contribute to STD risk and vulnerability. Data on sexual behaviors, condom use, substance use, healthcare access, and socioeconomic status help identify populations at the highest risk of STD acquisition and transmission and guide the development of tailored interventions and targeted prevention strategies.^[54]^ Furthermore, surveillance data on comorbidities, coinfections, and syndemics such as HIV/AIDS, viral hepatitis, and substance use disorders enable stakeholders to address the complex interactions between different health conditions and develop integrated approaches to care and prevention.

Surveillance and monitoring efforts also support international collaboration and knowledge sharing, facilitating data exchange, standardization of methodologies, and harmonization of surveillance practices across countries and regions. Through initiatives such as the Global AIDS Monitoring (GAM) system, the Global Gonococcal Antimicrobial Surveillance Program (GASP), and the WHO’s Global Health Observatory (GHO), stakeholders can access standardized data on STD epidemiology, trends, and responses, enabling cross-country comparisons, benchmarking, and evaluation of progress towards global targets and commitments.^[55]^

### 3.21. Resilience and adaptation

Resilience and adaptation are essential principles in the realm of STD prevention and control, emphasizing the ability of individuals, communities, and healthcare systems to respond effectively to changing circumstances, emerging threats, and unexpected challenges.^[56]^ In the face of evolving epidemiological trends, social determinants of health, and environmental factors, resilience and adaptation enable stakeholders to navigate uncertainty, overcome obstacles, and sustain progress toward reducing the burden of STDs and promoting sexual health and well-being.^[57]^

One key aspect of resilience and adaptation in STD prevention and control is the ability of healthcare systems to respond effectively to shifts in disease burden, transmission dynamics, and healthcare needs. By building flexible and responsive healthcare systems that adapt to changing epidemiological trends, stakeholders can ensure that essential STD prevention, testing, treatment, and support services remain accessible and available to all individuals, regardless of their circumstances.^[58]^ This may involve strengthening primary care infrastructure, integrating STD services with other health services, and adopting innovative service delivery models like telemedicine and mobile clinics to reach underserved populations and remote areas.

Furthermore, resilience and adaptation require a proactive approach to addressing emerging threats and vulnerabilities, such as antimicrobial resistance, changing sexual behaviors, and the impact of environmental factors such as climate change and urbanization. By monitoring trends in STD epidemiology, conducting risk assessments, and engaging in scenario planning, stakeholders can anticipate future challenges and develop proactive strategies to mitigate risks, prevent outbreaks, and protect the health of populations.^[59]^ This may involve strengthening surveillance systems, enhancing laboratory capacity, and investing in research and development of new diagnostics, treatments, and prevention technologies.

Community resilience and empowerment are also essential for effective STD prevention and control, enabling communities to mobilize resources, support each other, and advocate for their needs in the face of adversity. By fostering social cohesion, building support networks, and promoting community-led approaches to health promotion and advocacy, stakeholders can empower communities to address the social determinants of health, challenge stigma and discrimination, and create environments that promote sexual health and well-being for all individuals.^[60]^ This may involve supporting community-based organizations, providing training and capacity-building opportunities, and facilitating collaboration and knowledge sharing among diverse stakeholders.

Moreover, resilience and adaptation require a commitment to learning from past experiences, evaluating the effectiveness of interventions, and continuously improving strategies and approaches based on feedback and evidence. By fostering a culture of learning, innovation, and continuous improvement, stakeholders can identify gaps in services, address barriers to access, and optimize the impact of STD prevention and control efforts over time.^[61]^ This may involve conducting program evaluations, engaging in quality improvement initiatives, and sharing best practices and lessons learned with other stakeholders to inform future action.

### 3.22. Social and economic implications of STDs

The social and economic implications of STDs are profound and multifaceted, encompassing a wide range of impacts on individuals, families, communities, and societies as a whole. From the direct costs of healthcare services to the broader societal consequences of stigma, discrimination, and lost productivity, STDs have far-reaching effects that extend beyond the realm of public health.^[10]^

At the individual level, STDs can have significant physical, emotional, and financial consequences for affected individuals. The direct costs of seeking testing, treatment, and care for STDs can be substantial, particularly for those who lack health insurance or access to affordable healthcare services. Additionally, STD-related complications such as pelvic inflammatory disease, infertility, chronic pain, and certain types of cancer can have long-term health effects and impair individuals’ quality of life. Furthermore, the stigma and shame associated with STDs can lead to social isolation, relationship difficulties, and psychological distress, exacerbating the emotional toll of living with these infections.^[11]^

The economic burden of STDs extends beyond the individual level to impact families, communities, and societies as a whole. The costs of treating STDs, including medications, laboratory tests, and healthcare provider visits, impose a significant financial burden on healthcare systems and taxpayers. Moreover, the indirect costs of STDs, such as lost productivity due to illness, disability, and absenteeism from work or school, can have a substantial impact on economic productivity and growth. Additionally, the social and economic consequences of untreated STDs, such as reduced educational attainment, lower workforce participation, and higher rates of poverty, can perpetuate cycles of disadvantage and inequality within communities.^[12]^

Furthermore, the social and economic implications of STDs are often exacerbated by intersecting factors such as gender, race, ethnicity, sexual orientation, socioeconomic status, and geographic location. Marginalized populations, including racial and ethnic minorities, LGBTQ + individuals, sex workers, people who inject drugs, and adolescents, are disproportionately affected by STDs due to structural inequities such as poverty, lack of access to healthcare, discrimination, and stigma.^[13]^ These populations may face barriers to accessing sexual health services, experience higher rates of STD-related morbidity and mortality, and be more vulnerable to the social and economic consequences of living with STDs.

Addressing the social and economic implications of STDs requires a comprehensive and multi-sectoral approach that addresses the underlying determinants of health, promotes equity and social justice, and fosters collaboration among diverse stakeholders. This may involve expanding access to affordable healthcare services, implementing comprehensive sexual health education programs, promoting condom use and other prevention strategies, and reducing stigma and discrimination against affected individuals and communities.^[14]^ Additionally, investing in research, surveillance, and monitoring of STD trends and impacts can help inform evidence-based interventions and allocate resources effectively to address the social and economic burden of STDs.

### 3.23. Innovations in the treatment and care of STDs

In the ever-evolving landscape of STD prevention and control, innovations in treatment and care play a pivotal role in improving outcomes for individuals affected by these infections. From advancements in medication development to innovative approaches in patient-centered care, these innovations transform how STDs are diagnosed, treated, and managed, offering hope for better health outcomes and enhanced quality of life for affected individuals.^[15]^

One significant area of innovation in STD treatment is the development of new medications and treatment modalities for bacterial and viral infections. For bacterial STDs such as gonorrhea and syphilis, the emergence of antimicrobial resistance has posed a significant challenge to traditional treatment regimens. Researchers are also exploring alternative antibiotics, combination therapies, and novel treatments, such as phage therapy, to combat resistant strains and effectively manage these infections. Similarly, for viral STDs such as HIV and HSV, advances in antiviral medications, immune-based therapies, and gene editing technologies hold promise for controlling viral replication, reducing transmission risk, and improving long-term outcomes for affected individuals.^[16]^

In addition to medication development, diagnostic technology innovations are revolutionizing how STDs are detected and diagnosed. Traditional laboratory-based tests for STDs such as chlamydia and gonorrhea often require time-consuming sample collection, processing, and analysis, delaying diagnosis and treatment initiation. However, advances in point-of-care testing (POCT), molecular diagnostics, and rapid testing platforms enable real-time detection of STDs at the point of care, allowing for same-day diagnosis and treatment initiation.^[17]^ These rapid tests offer benefits such as increased convenience, reduced time to results, and improved patient satisfaction, facilitating timely intervention and reducing the risk of onward transmission.

Furthermore, innovations in patient-centered care are enhancing the delivery of STD services and improving the patient experience. Traditionally, STD care has been delivered in clinical settings such as hospitals, clinics, and private practices, which may be inaccessible or stigmatizing for specific populations. However, innovative approaches such as telemedicine, mobile health clinics, and community-based outreach programs are expanding access to STD services and reaching underserved populations where they are. Telemedicine platforms enable remote consultations, counseling, and prescription refills, eliminating barriers such as transportation and childcare and empowering individuals to take control of their sexual health from the comfort of their own homes.^[18]^

Moreover, advancements in holistic and integrated care models are addressing the complex needs of individuals affected by STDs, including their physical, emotional, and social well-being. Integrated care models, which combine STD services with other essential health and social services such as mental health care, substance use treatment, and social support, offer comprehensive support to individuals living with STDs and address the underlying determinants of health that contribute to STD risk and vulnerability.^[19]^ By addressing the social, behavioral, and structural factors that influence STD transmission and outcomes, these innovative care models promote holistic well-being and improve health outcomes for affected individuals and communities.

### 3.24. Psychosocial impacts and mental health

The psychosocial impacts of STDs extend far beyond the physical symptoms of infection, profoundly affecting the mental health and well-being of individuals and communities. From the moment of diagnosis to the long-term management of chronic infections, STDs can have a significant impact on emotional well-being, relationships, and overall quality of life.^[20]^

Upon receiving an STD diagnosis, individuals may experience a range of emotional responses, including shock, fear, shame, and guilt. The stigma associated with STDs, fueled by societal attitudes and misconceptions, can exacerbate these feelings and lead to social isolation, self-blame, and internalized stigma. Additionally, concerns about disclosure, rejection, and judgment from partners, family members, and peers can further compound the emotional burden of living with an STD.

The psychological impact of STDs can extend beyond the individual to affect interpersonal relationships and sexual behavior. For individuals in committed relationships, the disclosure of an STD diagnosis can pose challenges to trust, communication, and intimacy, leading to feelings of betrayal, resentment, and uncertainty about the future of the relationship. Similarly, for individuals who are dating or seeking new sexual partners, the fear of rejection or transmission can create barriers to forming intimate connections and lead to avoidance of sexual relationships altogether.^[21]^

Furthermore, the management of chronic STDs such as HIV/AIDS, HSV, and HPV can pose ongoing challenges to mental health and well-being. The daily regimen of medication adherence, regular medical appointments, and monitoring of viral load or disease progression can be emotionally taxing and disruptive to daily life. Additionally, the uncertainty about long-term health outcomes, the potential for disease progression, and the risk of transmitting the infection to others can create feelings of anxiety, depression, and existential distress.^[22]^

Moreover, the psychosocial impacts of STDs are often intertwined with broader social determinants of health, including socioeconomic status, race, ethnicity, gender identity, and sexual orientation. Marginalized populations, including racial and ethnic minorities, LGBTQ + individuals, and people living in poverty, may face additional barriers to accessing mental health support and coping resources, exacerbating disparities in mental health outcomes. Furthermore, intersecting forms of discrimination and oppression, such as racism, homophobia, and transphobia, can compound the emotional toll of living with an STD and contribute to feelings of social exclusion, marginalization, and identity-related stress.^[23]^

Addressing the psychosocial impacts of STDs requires a comprehensive and multidisciplinary approach that integrates mental health support, counseling, and psychosocial interventions into STD care and prevention programs. Providing individuals with access to confidential, non-judgmental counseling and support services can help alleviate feelings of shame, guilt, and isolation and empower individuals to navigate the emotional challenges of living with an STD. Additionally, promoting destigmatization and education about STDs can help reduce stigma and discrimination, foster supportive social networks, and create environments that promote acceptance, empathy, and understanding.^[2]^

### 3.25. Health disparities and health equity

Health disparities and health equity are critical issues in the realm of STDs, highlighting the unequal distribution of STD burden and access to care among different populations. A complex interplay of social, economic, and structural factors, including race, ethnicity, gender, sexual orientation, socioeconomic status, geographic location, and access to healthcare, influences these disparities.^[24]^

Racial and ethnic minorities, particularly Black, Hispanic/Latinx, and Indigenous communities, experience disproportionate rates of STDs compared to white populations. Structural inequities such as poverty, lack of access to healthcare, discrimination, and racism contribute to higher rates of STD transmission and poorer health outcomes among these populations. Additionally, historical and ongoing injustices, such as the legacy of slavery, colonization, and systemic racism, have created environments where individuals from marginalized racial and ethnic groups face increased vulnerability to STDs due to limited access to education, economic opportunities, and healthcare services.^[25]^

Gender disparities also play a significant role in shaping STD burden and access to care. Women, particularly young women and transgender women, are disproportionately affected by certain STDs, such as chlamydia and HPV, due to biological factors, gender-based violence, and unequal power dynamics in sexual relationships. Additionally, LGBTQ + individuals, including gay and bisexual men and transgender individuals, face higher rates of STDs compared to heterosexual individuals, often due to stigma, discrimination, and barriers to accessing culturally competent and affirming healthcare services.^[26]^

Socioeconomic status is a significant determinant of STD risk and vulnerability, with individuals living in poverty experiencing higher rates of STDs compared to those with higher incomes. Economic disparities limit access to preventive services such as STD testing, contraception, and healthcare coverage, exacerbating STD transmission and perpetuating cycles of disadvantage and inequality. Additionally, individuals experiencing homelessness, incarceration, or substance use disorders face unique challenges in accessing sexual health services.^[27]^ They may be at heightened risk of STD acquisition and transmission due to unstable housing, incarceration, and injection drug use.

Geographic disparities also contribute to variations in STD burden and access to care, with certain regions and communities experiencing higher rates of STDs compared to others. Rural communities, in particular, face challenges such as limited access to healthcare services, shortage of healthcare providers, and stigma related to seeking sexual health services. Additionally, urban areas with high population density and socioeconomic deprivation may also experience elevated rates of STDs due to factors such as poverty, overcrowded housing, and limited access to education and healthcare.^[28]^

Addressing health disparities and promoting health equity in STD prevention and control requires a multifaceted and intersectional approach that addresses the underlying determinants of health and prioritizes the needs of marginalized and underserved populations. This includes increasing access to culturally competent and linguistically appropriate sexual health services, implementing comprehensive sexuality education programs, expanding access to contraception and reproductive healthcare services, and addressing social determinants of health such as poverty, discrimination, and lack of access to education and economic opportunities.^[29]^

Furthermore, promoting community engagement, empowerment, and leadership among affected populations is essential for developing interventions responsive to diverse communities’ unique needs and priorities. This may involve partnering with community-based organizations, grassroots activists, and affected individuals to co-create and implement interventions that address the social, economic, and structural factors that contribute to STD risk and vulnerability.

### 3.26. Policy and legal frameworks

Policy and legal frameworks play a crucial role in shaping the landscape of STD prevention and control, influencing everything from access to healthcare services to the implementation of prevention strategies and the protection of individuals’ rights. These frameworks encompass a wide range of laws, regulations, policies, and guidelines at the local, national, and international levels, aimed at addressing STDs from a public health and human rights perspective.

At the national level, governments develop policies and laws to guide STD prevention and control efforts, often in collaboration with public health agencies, healthcare providers, community organizations, and other stakeholders. These policies may include guidelines for STD testing and treatment, regulations for reporting STD cases to public health authorities, and requirements for healthcare providers to offer STD counseling, testing, and treatment to their patients. Additionally, national governments may allocate funding and resources to support STD prevention programs, research initiatives, and public awareness campaigns aimed at reducing STD transmission and promoting sexual health.^[30]^

Furthermore, legal frameworks play a critical role in protecting the rights and well-being of individuals affected by STDs, ensuring access to confidential and nondiscriminatory healthcare services, and preventing discrimination based on STD status. Laws such as the Health Insurance Portability and Accountability Act (HIPAA) in the United States, for example, protect the privacy and confidentiality of individuals’ health information, including STD test results and medical records. Similarly, anti-discrimination laws prohibit discrimination based on STD status in employment, housing, education, and healthcare settings, ensuring that individuals living with STDs are not unfairly stigmatized or denied access to essential services.

Internationally, organizations such as the WHO and the United Nations (UN) develop guidelines and recommendations for STD prevention and control, which member states can adopt and implement within their own legal and policy frameworks. These international guidelines help ensure consistency and harmonization of STD prevention strategies across countries and regions, facilitate knowledge sharing and collaboration among stakeholders, and promote a coordinated global response to the STD epidemic.^[31]^

Moreover, legal frameworks also play a role in regulating behaviors and practices that contribute to STD transmission, such as sex work, drug use, and sexual violence. Laws and regulations related to these activities can have a significant impact on STD risk and vulnerability, influencing access to prevention services, harm reduction interventions, and legal protections for marginalized populations. In some cases, laws criminalizing certain behaviors, such as sex work or drug possession, can exacerbate stigma, drive these activities underground, and hinder efforts to reach at-risk populations with essential health services.

Addressing the complex legal and policy issues surrounding STD prevention and control requires a multi-sectoral and evidence-based approach that balances public health objectives with respect for individual rights and autonomy. This may involve conducting policy analysis and advocacy to identify gaps in existing legal frameworks, promote reforms that support STD prevention and care, and challenge laws and regulations that perpetuate stigma, discrimination, and barriers to access. Additionally, engaging with affected communities, civil society organizations, and policymakers can help ensure that legal and policy responses to STDs are responsive to the diverse needs and priorities of all individuals and communities affected by these infections.^[32]^

### 3.27. Ethical Considerations

Ethical considerations are paramount in the realm of STD prevention and control, guiding decision-making, policy development, and practice in ways that prioritize the rights, dignity, and well-being of individuals affected by STDs. From ensuring confidentiality and informed consent to promoting autonomy and justice, ethical principles inform every aspect of STD care, research, and public health interventions.

One fundamental ethical principle in STD prevention and control is respect for individual autonomy and informed consent. Individuals have the right to make informed decisions about their sexual health, including whether to undergo STD testing, treatment, and disclosure of their STD status to partners or healthcare providers. Healthcare providers must provide accurate information about STDs, treatment options, and potential risks and benefits, allowing individuals to make autonomous decisions that align with their values, preferences, and circumstances. Additionally, ensuring the confidentiality and privacy of an individual’s health information is essential for fostering trust and maintaining the integrity of the patient-provider relationship.^[33]^

Another key ethical consideration is the principle of beneficence, which requires healthcare providers and public health practitioners to act in the best interests of their patients and the broader community. This may involve providing timely and appropriate STD testing, treatment, and counseling services to individuals affected by STDs, regardless of their ability to pay or access to healthcare. Additionally, promoting preventive interventions such as vaccination, condom use, and harm reduction strategies can help reduce the risk of STD transmission and protect the health and well-being of individuals and communities.^[34]^

Furthermore, the principle of non-maleficence underscores the importance of avoiding harm and minimizing risks to individuals affected by STDs. This may involve ensuring that STD testing, treatment, and prevention services are provided in a culturally competent, non-judgmental, and respectful manner, free from stigma, discrimination, and coercion. Additionally, healthcare providers must minimize the risk of adverse outcomes associated with STDs, such as complications, transmission to partners, and psychosocial distress, through appropriate medical management, counseling, and support services.^[35]^

Justice is another ethical principle central to STD prevention and control efforts, emphasizing fairness, equity, and inclusivity in distributing resources and access to care. Addressing health disparities and promoting health equity requires a commitment to addressing the underlying social determinants of health that contribute to STD risk and vulnerability, including poverty, discrimination, and lack of access to education and healthcare services. Additionally, ensuring that STD prevention and control efforts are accessible, affordable, and culturally relevant for all individuals and communities, particularly those who are marginalized or underserved, is essential for promoting justice and reducing health inequities.^[36]^

Moreover, ethical considerations extend to research involving STDs, including issues related to informed consent, privacy, and protection of human subjects. Researchers are responsible for obtaining informed consent from study participants, protecting their privacy and confidentiality, and minimizing risks and burdens associated with participation in research studies. Additionally, researchers must ensure that study findings are disseminated transparently and responsibly, contributing to advancing knowledge and informing evidence-based interventions and policies.

### 3.28. Global health security and pandemic preparedness

Global health security and pandemic preparedness are critical considerations in the context of STDs, highlighting the interconnectedness of STD prevention and control efforts with broader efforts to prevent and respond to emerging infectious diseases and public health threats. While STDs may not always be at the forefront of discussions on global health security, they pose significant challenges to health systems, economies, and social well-being, particularly in the context of pandemics such as HIV/AIDS and COVID-19.

One of the key lessons learned from past pandemics, such as HIV/AIDS, is the importance of early detection and rapid response to emerging infectious diseases. STD surveillance systems play a vital role in monitoring trends in STD prevalence, incidence, and antimicrobial resistance, providing early warning signals for potential outbreaks and informing targeted interventions to control transmission by integrating STD surveillance into broader infectious disease surveillance systems, public health authorities can detect and respond to emerging threats more effectively, preventing the spread of STDs and reducing their impact on population health.^[37]^

Furthermore, the HIV/AIDS pandemic has underscored the importance of community engagement, empowerment, and leadership in pandemic response efforts. Community-based organizations, grassroots activists, and affected individuals played a crucial role in advocating for access to HIV testing, treatment, and prevention services, challenging stigma and discrimination, and mobilizing resources to support those affected by the epidemic. Similarly, in the context of STD prevention and control, empowering communities to take ownership of their sexual health, advocate for their needs, and participate in decision-making processes is essential for building resilient and responsive health systems.^[38]^

Additionally, pandemic preparedness efforts must address the intersecting vulnerabilities and social determinants of health that contribute to STD risk and vulnerability, particularly among marginalized and underserved populations. Addressing issues such as poverty, lack of access to healthcare, discrimination, and stigma is essential for reducing health disparities and promoting health equity in STD prevention and control efforts. Moreover, integrating STD prevention and control into broader health systems strengthening initiatives can help build resilience and capacity to respond to emerging infectious diseases and other public health threats, ensuring a more holistic and sustainable approach to global health security.^[39]^

In the context of the COVID-19 pandemic, STD prevention and control efforts have faced unique challenges and opportunities. The pandemic has disrupted healthcare services, including STD testing, treatment, and prevention programs, leading to disruptions in care and increased risk of STD transmission. However, the COVID-19 pandemic has also highlighted the importance of innovative approaches such as telemedicine, mobile health clinics, and self-testing kits for expanding access to STD services and reaching underserved populations where they are. Additionally, the pandemic has underscored the need for resilient and adaptable health systems that can respond effectively to emerging threats, prioritize the needs of vulnerable populations, and promote equity and justice in healthcare delivery.

### 3.29. Diagnosis and management of STDs

Diagnosis and management are crucial aspects of effectively addressing STDs, ensuring timely detection, appropriate treatment, and prevention of further transmission. Healthcare providers employ a variety of approaches and techniques to diagnose STDs accurately and manage them effectively, tailored to the specific characteristics of each infection and the individual needs of patients.^[1]^

One of the primary methods for diagnosing STDs is through laboratory testing of biological specimens, such as blood, urine, genital swabs, or tissue samples. These tests can detect the presence of pathogens, antibodies, or genetic material associated with STDs, allowing for accurate diagnosis and targeted treatment. Common laboratory tests for STDs include nucleic acid amplification tests (NAATs), enzyme immunoassays (EIAs), polymerase chain reaction (PCR) assays, and culture-based methods, each with its advantages and limitations depending on the specific pathogen being targeted.^[2]^

In addition to laboratory testing, healthcare providers may use clinical examination and history-taking to diagnose STDs, particularly infections with characteristic symptoms or signs. Physical examination may involve visual inspection of the genital area, palpation of lymph nodes, and evaluation of mucosal surfaces for lesions, discharge, or other abnormalities. Obtaining a thorough sexual history, including information about sexual practices, partners, and risk factors, is also essential for guiding diagnostic and management decisions and identifying individuals at increased risk of STD acquisition.^[3]^

Once a diagnosis is made, the management of STDs typically involves a combination of pharmacological treatment, behavioral counseling, partner management, and preventive interventions. Pharmacological treatment may include antibiotics, antiviral medications, or other antimicrobial agents, depending on the type of STD and the susceptibility of the causative pathogen. Behavioral counseling aims to promote safer sexual practices, enhance adherence to treatment regimens, and reduce the risk of STD transmission to partners or reinfection. Partner management involves notifying and treating sexual partners of individuals diagnosed with STDs to prevent the further spread of infection and reduce the risk of complications.^[39]^

Moreover, preventive interventions play a critical role in managing STDs and reducing their impact on individuals and communities. These interventions may include vaccination against STDs such as HPV, HBV, and certain strains of meningococcus and pneumococcus, which can prevent acquiring and transmitting these infections. Additionally, promoting condom use, regular STD testing, pre-exposure prophylaxis (PrEP) for HIV prevention, and harm reduction strategies for individuals at increased risk of STD acquisition, such as people who inject drugs or engage in transactional sex, can help reduce transmission and improve health outcomes.^[40]^

In essence, treating STIs often involves the use of antimicrobial drugs to eradicate the causative pathogens and alleviate symptoms. These drugs target the specific mechanisms of action of the pathogens, inhibit their growth or replication, and ultimately help restore the individual’s health and prevent further transmission of the infection.^[40]^

One commonly used class of antimicrobial drugs for STIs is antibiotics, which are effective against bacterial infections such as chlamydia, gonorrhea, and syphilis. For example, azithromycin and doxycycline are frequently prescribed antibiotics for the treatment of chlamydia infections. Azithromycin inhibits bacterial protein synthesis, while doxycycline disrupts bacterial protein production by binding to the ribosome. These antibiotics have relatively long half-lives, with azithromycin having a half-life of approximately 68 hours and doxycycline ranging from 12 to 25 hours. A typical course of treatment for chlamydia infection involves a single dose of azithromycin or a week-long course of doxycycline.^[32]^

For gonorrhea, the recommended treatment often involves a combination of antibiotics to combat increasing antimicrobial resistance. Ceftriaxone, a third-generation cephalosporin, is commonly used with azithromycin or doxycycline to treat gonorrhea infections. Ceftriaxone inhibits bacterial cell wall synthesis, while azithromycin and doxycycline target bacterial protein synthesis. Ceftriaxone has a half-life of approximately 5 to 9 hours and is typically administered as a single intramuscular injection, while azithromycin and doxycycline are administered orally.^[54]^

For syphilis, penicillin remains the drug of choice for treatment, particularly for primary and secondary syphilis infections. Penicillin disrupts bacterial cell wall synthesis, ultimately leading to cell death. The duration of treatment for syphilis varies depending on the stage of the infection, with early-stage infections typically requiring a single intramuscular injection of benzathine penicillin G and more advanced infections necessitating multiple doses over a longer duration.^[58]^

In addition to antibiotics, antiviral drugs are used to treat viral STIs, such as HSV and HIV. Antiviral drugs such as acyclovir, valacyclovir, and famciclovir are commonly prescribed for HSV infections to reduce the frequency and severity of recurrent outbreaks and alleviate symptoms. These antiviral drugs work by inhibiting viral DNA synthesis and replication, ultimately suppressing viral activity and reducing the duration and severity of symptoms. They have relatively short half-lives, with acyclovir having a half-life of approximately 2.5 to 3.3 hours and valacyclovir and famciclovir being converted to acyclovir in the body.^[59]^

For HIV/AIDS, combination antiretroviral therapy (ART) is the standard of care for treatment, consisting of a combination of drugs from different classes to suppress viral replication and restore immune function. These drugs include nucleoside reverse transcriptase inhibitors, non-nucleoside reverse transcriptase inhibitors, protease inhibitors, integrase strand transfer inhibitors, and entry inhibitors. ART targets different stages of the HIV life cycle, preventing the virus from replicating and reducing viral load in the body.^[61]^ The duration of treatment with ART is lifelong, as discontinuation of therapy can lead to viral rebound and disease progression. Table 2 presents clinical guidelines for the management of STDs.

**Table 2:** A broad table summarizing essential clinical guidelines for the management of STDs from various national and international organizations.

Organization	Guideline name	Focus areas	Highlights
Centers for Disease Control and Prevention	STD Treatment Guidelines	Diagnosis, treatment, and management of STDs	- Evidence-based recommendations for STD testing, treatment regimens, partner management, and prevention strategies - Regularly updated to reflect changes in STD epidemiology and clinical practice
World Health Organization	Guidelines for the Management of Sexually Transmitted Infections	Diagnosis, treatment, and prevention of sexually transmitted infections worldwide	- Global recommendations for syndromic management of STIs, laboratory diagnosis, antimicrobial resistance, and prevention strategies such as condom use and vaccination against HPV and hepatitis B
European Centre for Disease Prevention and Control	Guidelines for the Management of Chlamydia trachomatis Infections	Diagnosis, treatment, and partner management of chlamydia infections	- Recommendations for diagnosis, treatment, and partner management of chlamydia infections, as well as strategies for prevention and control at the population level
British Association for Sexual Health and HIV	British Association for Sexual Health and HIV Guidelines	Diagnosis, treatment, and management of sexually transmitted infections in the United Kingdom	- Clinical guidelines developed by expert panels of healthcare professionals in the UK. - Cover a wide range of STDs and related conditions
International Antiviral Society-USA	Guidelines for the Management of HIV and Other Viral Infections	Management of HIV/AIDS and viral infections commonly associated with HIV	- Recommendations for managing viral infections such as HSV and HPV - Widely regarded as authoritative in the field of HIV/AIDS

These guidelines serve as valuable resources for healthcare providers, policymakers, and public health officials, providing evidence-based recommendations for diagnosing, treating, and preventing STDs. By standardizing clinical practice and promoting consistency in care, these guidelines contribute to improved health outcomes for individuals affected by STDs and support efforts to reduce the burden of STDs at the population level.

HIV = human immunodeficiency virus, HSV = herpes simplex virus, HPV = human papillomavirus, STDs = sexually transmitted diseases.

## 4. Conclusion

The global burden of STDs represents a significant contribution to the field of public health and international health planning. By synthesizing the latest evidence and employing rigorous methodology, we have gained valuable insights into the magnitude and implications of STDs worldwide.

Our findings underscore the substantial impact of STDs on individuals, communities, and healthcare systems, highlighting the urgent need for effective prevention and control strategies. Through the quantification of DALYs and the identification of high-risk populations and geographic areas, we have provided a nuanced understanding of the distribution and determinants of STD burden.

Importantly, our review fills existing gaps in the literature by offering evidence-based recommendations for improving STD prevention and control efforts. By evaluating the effectiveness of current strategies and identifying areas for enhancement, we have paved the way for more targeted interventions and resource allocation.

The implications of our study extend beyond academic discourse, directly informing global health planning initiatives aimed at reducing the burden of STDs and improving sexual health outcomes. Our findings call for a multi-sectoral approach that integrates surveillance, education, screening, treatment, and community engagement to address the complex challenges of STDs.

Moving forward, policymakers, healthcare practitioners, and public health officials must heed the insights provided by our review and prioritize investment in evidence-based interventions. By working collaboratively and leveraging the evidence presented, we can make meaningful strides towards achieving the Sustainable Development Goals related to sexual and reproductive health.

Our review catalyzes action, empowering stakeholders to make informed decisions and mobilize resources effectively to combat the global burden of STDs. Together, we can create a world where all individuals have access to comprehensive sexual health services and enjoy lives free from the devastating impact of STDs.

## Acknowledgments

We sincerely thank all individuals and organizations who contributed to completing this manuscript on “Global Perspectives on the Burden of Sexually Transmitted Diseases.”

First and foremost, we thank the participants who generously shared their time and insights; the search would not have only helped them. Your contributions are invaluable in advancing our understanding of the global burden of sexually transmitted diseases and informing public health initiatives.

We thank the research team members for their dedication and hard work in data collection, analysis, and manuscript preparation. Your commitment to excellence has ensured the quality and rigor of this study.

We also extend our appreciation to the reviewers for their constructive feedback and valuable suggestions, which have helped improve the clarity and impact of this manuscript.

Lastly, we thank our families, friends, and colleagues for their encouragement and understanding throughout this endeavor. Your unwavering support has been a source of strength and inspiration.

Thank you to all who have contributed to this work. Your dedication and collaboration have been essential in bringing this manuscript to fruition.

## Author contributions

**Conceptualization:** Chukwuka Elendu, Dependable C. Amaechi, Ijeoma D. Elendu, Tochi C. Elendu, Emmanuel C. Amaechi.

**Data curation:** Chukwuka Elendu, Dependable C. Amaechi, Ijeoma D. Elendu, Tochi C. Elendu, Emmanuel C. Amaechi.

**Formal analysis:** Chukwuka Elendu, Dependable C. Amaechi, Ijeoma D. Elendu, Tochi C. Elendu, Emmanuel C. Amaechi.

**Funding acquisition:** Chukwuka Elendu, Dependable C. Amaechi, Ijeoma D. Elendu, Tochi C. Elendu, Emmanuel C. Amaechi.

**Investigation:** Chukwuka Elendu, Dependable C. Amaechi, Ijeoma D. Elendu, Tochi C. Elendu, Emmanuel C. Amaechi.

**Methodology:** Chukwuka Elendu, Dependable C. Amaechi, Ijeoma D. Elendu, Tochi C. Elendu, Emmanuel C. Amaechi.

**Project administration:** Chukwuka Elendu, Dependable C. Amaechi, Ijeoma D. Elendu, Tochi C. Elendu, Emmanuel C. Amaechi, Emmanuel U. Usoro, Nkechi L. Chima-Ogbuiyi, Divine B. Arrey Agbor, Chukwunnonso J. Onwuegbule, Eniola F. Afolayan, Benjamin B. Balogun.

**Resources:** Chukwuka Elendu, Dependable C. Amaechi, Ijeoma D. Elendu, Tochi C. Elendu, Emmanuel C. Amaechi, Emmanuel U. Usoro, Nkechi L. Chima-Ogbuiyi, Divine B. Arrey Agbor, Chukwunnonso J. Onwuegbule, Eniola F. Afolayan, Benjamin B. Balogun.

**Software:** Chukwuka Elendu, Dependable C. Amaechi, Ijeoma D. Elendu, Tochi C. Elendu, Emmanuel C. Amaechi, Emmanuel U. Usoro, Nkechi L. Chima-Ogbuiyi, Divine B. Arrey Agbor, Chukwunnonso J. Onwuegbule, Eniola F. Afolayan, Benjamin B. Balogun.

**Supervision:** Chukwuka Elendu, Dependable C. Amaechi, Ijeoma D. Elendu, Tochi C. Elendu, Emmanuel C. Amaechi, Emmanuel U. Usoro, Nkechi L. Chima-Ogbuiyi, Divine B. Arrey Agbor, Chukwunnonso J. Onwuegbule, Eniola F. Afolayan, Benjamin B. Balogun.

**Validation:** Chukwuka Elendu, Dependable C. Amaechi, Ijeoma D. Elendu, Tochi C. Elendu, Emmanuel C. Amaechi, Emmanuel U. Usoro, Nkechi L. Chima-Ogbuiyi, Divine B. Arrey Agbor, Chukwunnonso J. Onwuegbule, Eniola F. Afolayan, Benjamin B. Balogun.

**Visualization:** Chukwuka Elendu, Dependable C. Amaechi, Ijeoma D. Elendu, Tochi C. Elendu, Emmanuel C. Amaechi, Emmanuel U. Usoro, Nkechi L. Chima-Ogbuiyi, Divine B. Arrey Agbor, Chukwunnonso J. Onwuegbule, Eniola F. Afolayan, Benjamin B. Balogun.

**Writing – original draft:** Chukwuka Elendu, Dependable C. Amaechi, Ijeoma D. Elendu, Tochi C. Elendu, Emmanuel C. Amaechi, Emmanuel U. Usoro, Nkechi L. Chima-Ogbuiyi, Divine B. Arrey Agbor, Chukwunnonso J. Onwuegbule, Eniola F. Afolayan, Benjamin B. Balogun.

**Writing – review & editing:** Chukwuka Elendu, Dependable C. Amaechi, Ijeoma D. Elendu, Tochi C. Elendu, Emmanuel C. Amaechi, Emmanuel U. Usoro, Nkechi L. Chima-Ogbuiyi, Divine B. Arrey Agbor, Chukwunnonso J. Onwuegbule, Eniola F. Afolayan, Benjamin B. Balogun.
